# Improving Maternity Care in India’s Private Hospitals: Quality Certification? Yes, but More Is Needed

**DOI:** 10.9745/GHSP-D-22-00514

**Published:** 2022-12-21

**Authors:** Matthews Mathai

**Affiliations:** a Associate Editor, *Global Health: Science and Practice* Journal; Independent consultant, St. John’s, NL, Canada.

See related article by Marx Delaney et al.

Over the last decade, the focus in maternal and newborn health care has shifted from improving coverage of health care services toward ensuring that care provided through these services is of the best quality. Quality of care encompasses the provision of care as well as the experience of care.^1^ Global standards for quality maternal and newborn care published by the World Health Organization^2^ have been widely adopted by national governments. Many partnerships and networks have been formed to promote the quality-of-care agenda, particularly in low- and middle-income countries.^3^

Marx Delaney et al. report on a quality improvement initiative in private hospitals in India.^4^ Among the 24 million births in India every year, 94% of those in urban areas and 88% of those in rural areas take place in health care facilities. More women (65%) in rural areas than in urban areas (53%) give birth in public health care facilities.^5^ The remainder of institutional births take place in private hospitals, which are independently managed and less often subject to critical regulatory oversight.

Manyata is a quality improvement and certification initiative offered by the Federation of Obstetric and Gynaecological Societies of India (FOGSI) for private facilities providing maternal care.^6^ FOGSI is an umbrella organization with more than 38,000 members spread through 263 societies across the country working mostly in private facilities. Manyata’s vision is to “ensure all women have access to safer and respectful care during and after childbirth in India,” and it aims to “improve quality of maternity and newborn care services in private facilities by training doctors, nursing and administrative staff on essential clinical, facility and patient care protocols.”

The Manyata initiative, which several national and international partners supported, was initially implemented in 3 states in India but is now being implemented in other states. It focuses on mentorship and clinical standards to improve health care providers’ knowledge, skills, and adherence to key childbirth-related clinical practices. Interested private hospitals owned, managed, or staffed by FOGSI members apply for certification under the initiative. Certification is expected to act “as a stamp of quality ensuring consistent, safe and respectful maternal care for women during and immediately after childbirth.”

Marx Delaney et al.^4^ report on the secondary analysis of data collected between October 2016 and February 2019 from private hospitals in the first 3 participating states. The analyses included comparisons of knowledge and skills assessment scores obtained before and immediately after training and the proportion of hospitals that passed quality standards at baseline and endline. The authors also assessed changes in pregnancy outcomes using autoregressive modeling. Overall, there were improvements in knowledge and skills immediately after training and better adherence to selected standards at endline assessment. However, there were no significant changes in reported complications from these facilities.

FOGSI and its partners should be complimented for the initiative. However, there are some areas where the initiative could be strengthened.

Many nurses in smaller private hospitals are poorly trained in childbirth-related clinical practices, if at all, and 2–3 days may be insufficient to bring them up to speed. Most hospitals (59%) that participated in the initiative had fewer than 30 births every month and hence, fewer potential complications. Knowledge and skills gained will be lost rapidly if the opportunities to practice or refresh these competencies are not readily available. In a hierarchical system, such as in India, where doctors make most decisions on clinical management, training nurses alone may not be sufficient to impact the quality of care and outcomes. In hospitals that have many doctors, getting consensus on common care pathways, even where evidence-based guidelines exist, can be challenging.

Manyata includes 16 standards of quality care, but a hospital is required to achieve only 14 standards for certification under the initiative. Furthermore, for certification, the bars have been set relatively low—it is sufficient if evidence of adherence to standards was noted in 50% of medical records or 50% of patient interviews. The fact that the cesarean delivery standard was achieved by only 9% of the private hospitals is of particular concern because cesarean deliveries account for 47% of births in private facilities but only for 14.3% in public facilities.^5^

Improving data collection on clinical practices and outcomes was not a requirement of the initiative. Some hospitals provided data, but others did not. While the authors report that referral rates for preeclampsia/eclampsia and newborn with sepsis fell after the intervention, there is no information on the health outcomes of women and babies with these problems. Health care providers’ reluctance to share outcome data is understandable, especially in places where these data could be used for punitive purposes. However, professional organizations, such as FOGSI, should work with their members to raise their awareness of the key role of data in improving quality of care. The Kerala Federation of Obstetrics and Gynaecology^7^ is a good example of a FOGSI member society in which obstetricians working in public and private facilities have successfully conducted confidential maternal death reviews for more than a decade.

Certification strategies can be effective in improving quality of care if training and mentoring support are provided and if there is periodic monitoring of processes and outcomes. Staff turnover can be high, and new or refresher training may be required. Certified facilities should demonstrate adherence to all standards—not only some of them—and the minimum score required to achieve any standard should be set significantly higher than the present 50%. Moving forward, FOGSI and partners should consider training the entire team that provides maternity care, not just individual cadres, and introducing periodic audits of clinical practices and outcomes as part of this initiative. FOGSI may also consider focusing more on improving quality of care in hospitals with more staff and more births, where the likelihood of complications and the potential for impact of the initiative are higher.
